# Blocking the interaction between S100A9 protein and RAGE V domain using S100A12 protein

**DOI:** 10.1371/journal.pone.0198767

**Published:** 2018-06-14

**Authors:** Revansiddha Katte, Chin Yu

**Affiliations:** Department of Chemistry, National Tsing Hua University, Hsinchu, Taiwan; Russian Academy of Medical Sciences, RUSSIAN FEDERATION

## Abstract

The proteins S100A9 and S100A12 are associated with the human S100 calcium-binding protein family. These proteins promote interaction with target proteins and alter their conformation when they bind to calcium ions in EF-hand motifs. The V domain of RAGE (Receptor for Advanced Glycation End products) is crucial for S100A9 binding. The binding of RAGE with S100 family proteins aids in cell proliferation. In this report, we demonstrate that S100A12 protein hinders the binding of S100A9 with the RAGE V-domain. We used fluorescence and NMR spectroscopy to analyze the interaction of S100A9 with S100A12. The binary complex models of S100A9-S100A12 were developed using data obtained from ^1^H-^15^N HSQC NMR titrations and the HADDOCK program. We overlaid the complex models of S100A9-S100A12 with the same orientation of S100A9 and the RAGE V-domain. This complex showed that S100A12 protein blocks the interaction between S100A9 and the RAGE V-domain. It means S100A12 may be used as an antagonist for S100A9. The results could be favorable for developing anti-cancer drugs based on S100 family proteins.

## 1. Introduction

The interaction mechanisms of S100 family proteins could be useful for inhibiting their interaction in humans with the V-domain of RAGE (Receptor for Advanced Glycation End products). It was reported that the human S100A12 protein interacted with S100A9 protein [1]. S100A9 and S100A12 belong to a family of more than 20 calcium-binding proteins with intracellular EF-hand motif and low molecular weight [2–4]. The S100 protein family is the largest group of calcium-binding proteins. S100A9 and S100A12 are phagocyte-specific S100 family members that include the group of calgranulins. These proteins expression patterns are a major source of myeloid origin cells, which could be advantageous for identifying these proteins [5]. These proteins are also called calprotectin proteins, which mostly occur in high concentrations within sites of inflammation associated with cancers [6], cystic fibrosis [7], rheumatoid arthritis [8], and other diseases [9].

The human S100A9 protein (also called L1 heavy chain, MRP-14, and calgranulin B) has a molecular weight of 13.2 kDa and contains a total of 114 amino acids [10, 11]. This amino acid sequence homology shows high similarity to the phagocyte-specific proteins S100A12 (46%) and S100A8 (30%). A few studies have predicted that S100A9 protein is one of the core contributors in the cardiovascular system during atherosclerosis and cardiac dysfunction [12], and that it regulates the amassing of neutrophils, monocytes, or macrophages [13], as well as cytokine production [14] and cell proliferation [15]. S100A9 is one of several proteins known as “damage-associated molecular pattern” (DAMP) proteins [16]. DAMP proteins activate signaling cascade pathways in multiple human diseases through interactions with target proteins, including RAGE and Toll-like receptor 4 (TLR4) [17,18].

RAGE has been a crucial therapeutic target due to its impact and relevance in a diverse range of human diseases and tumor growth [19–21]. The immunoglobulin superfamily includes an extracellular domain of RAGE that is a multi-ligand cell surface receptor [22, 23]. The ligands involve AGE, high mobility group box-1 (HMGB1), S100/calgranulins, beta-sheet fibrils and amyloid beta peptide. Moreover, S100A9, S100A11, S100A13, and S100P were observed to interact with RAGE to initiate signal transduction [24, 25].

We have determined the binding constant (K_*d*_) of the S100A9-S100A12 heterodimer complex by fluorescence spectroscopy. The HADDOCK (High Ambiguity Driven Protein-Protein Docking) program was used to examine the residues at the interfaces of two proteins which obtained from NMR (^1^H-^15^N HSQC) titrations [26]. Furthermore, S100A12 was identified to be an inhibitor that could interact with S100A9 and block the interface of S100A9 with the V-domain of RAGE. These bindings are demonstrated by the results of NMR HSQC titrations, fluorescence experiments, and HADDOCK calculation.

## 2. Materials and methods

### 2.1 Materials

Luria broth was obtained from APOLO Biochemical, while isotope-labeled ammonium chloride (^15^NH_4_Cl) and deuterium oxides (D_2_O) were obtained from Sigma-Aldrich. cDNA of S100A12 and S100A9 were supplied by Mission Biotech Company.

### 2.2. Preparation of the S100A12 and S100A9

The Human S100A12 protein was over expressed in *Escherichia coli* BL21 (DE3) strain using the pET-21b vector. The expression and purification of S100A12 was achieved using the previously described protocol [27]. The purified protein fraction was dialysed against NMR buffer (2 mM CaCl_2_, 50 mM Tris-HCl, 100 mM NaCl, 5 mM DTT, 1 mM EGTA, and 10% D_2_O, pH 7); and sample used for NMR spectroscopy ^15^N-labeled S100A12.

The human S100A9 protein was over expressed in the pET-21b vector using BL21 (DE3) as host cells. The expression and purification steps were followed as described previously [28]. The purified protein fraction was dialysed against NMR buffer (2 mM CaCl_2_, 50 mM Tris-HCl, 100 mM NaCl, 5 mM DTT, 1 mM EGTA, and 10% D_2_O, pH 7); and sample used for NMR spectroscopy ^15^N-labeled S100A9. SDS- PAGE analysis showed that the S100A12 and S100A9 protein samples were more than 95% pure (S1 and S2 Figs). Their molecular weights were verified using ESI-MS (S3 and S4 Figs).

### 2.3 NMR HSQC titration experiments

All NMR titrations were performed on a 700 MHz (Varian) NMR spectrometer at 298 K using cryogenic probes. All Protein samples were prepared using a same NMR buffer. The assignments for backbone and side-chain of S100A12 are available from the Biological Magnetic Resonance Bank (BMRB code: 19293) [27] for specific buffer conditions (100 mM NaCl, 0.02% (w/v) NaN_3_ and 10 mM Hepes, pH 6.5). The assignments for S100A9 in other buffer conditions (50 mM Tris-HCl, 2mM CaCl_2_, 100 mM NaCl, 10% D_2_O pH 7.5; BMRB code: 30017) [28]. The HSQC spectra were compared with these data to assign cross-peaks in our study.

The NMR titration experiments were carried out by adding unlabeled S100A9 to ^15^N-labeled S100A12 protein solution to the molar ratios 1:1. These proteins are exist as homodimer, so the proteins were first denatured with 8 M urea before associate as heterodimer or destabilized the quaternary structure with calcium-binding [29, 30, 31]. The urea was washed away with water, and the water was replaced with NMR buffer. In additionally, similar approach was used for reverse titration. The reverse titration (^15^N-labeled S100A9 with unlabeled S100A12 protein) was carried out at molar ratios of 1:0 and 1:1. We superimposed the HSQC titration spectra of the different ratios (1:0 and 1:1) for the reorganization of certain residues based on perturbed chemical shifts or diminished intensity at the crossing point of the two molecules. The spectra were examined using Sparky [32].

### 2.4 Molecular docking

HADDOCK is a commercially available platform for observing and analyzing intermolecular protein—protein interaction. HADDOCK version 2.2 was used to observe the docking of the S100A9 and A100A12 heterodimer complex structure. The structural coordinates of the S100A9 and S100A12 were acquired from the Protein Data Bank (PDB) (IDs: 5I8N and 2m9g, respectively). The residues of peculiar intensity decreases or perturbations were identified by ^1^H-^15^N HSQC NMR. These residues were used as input parameters for AIR (Ambiguous Interaction Constraints) at the binding sites. Among these residues, we considered the ones with a higher solvent accessibility to serve as the main interface residues. Therefore, those have a relative solvent accessibility (RSA) higher than 30% are defined as the active residues while those lower than 30% are the passive residues for the parameterization of HADDOCK. Here, RSA was calculated by the software NACCESS [33]. Every results of S100A9-S10012 complex was interconnected with a sum of energy comprising terms van der Waals, electrostatic, desolvation, restraint violation and buried surface area based on OPLS forcefield [34]. HADDOCK score was obtained by the following equation.

$$HADDOCK\;Score\;=\;1.0*E_{vdw\;}+0.2*E_{elec}+\;1.0*E_{desol}\;+0.1*E_{air}$$ (1)

Where E_vdw_ represents the intermolecular van der Waals energy; E_elec_ represents the intermolecular electrostatic energy; E_desol_ is the empirical desolvation energy term adapted from Fernandez [35]; whereas E_air_ represents energy imposed by ambiguous interaction restraint (AIR).

Approximately 2,000 structures were discerned from the standard HADDOCK procedure with the optimized potential for liquid simulation parameters. The residues on interface (having heavy atoms inside 5 Angstrom long from the nest protein) obtained around 200 complexes. These 200 complexes (out of 2000) having the lowest energy (or HADDOCK score) were used for semi-flexible simulated annealing process. With the help of short MD simulations in explicit water model (TIP3P) having 8-Angstrom hydration shell. Further, theses structures were grouped into 10 clusters based on the similarity in the constituent docking pose interface. A detailed analysis of the visualization and structural representations of first cluster was accomplished using PyMOL [36].

### 2.5 Fluorescence experiments for binding affinity (K_d_)

Fluorescence experiments are useful for calculating the dissociation constant of protein—protein interaction [37]. The Fluorescence experiments were conducted using a fluorescence spectrophotometer (Hitachi F-2500). The human S100A9 has one tryptophan residue that could be excited and emit fluorescence. Here we used 295 nm wavelength as a excitation light source and subsequent changes in the emission spectra were observed in the range 310 to 405 nm with a 5 nm slit width. Increasing concentrations of human S100A12 protein solution (0.4–7 μM) were added to S100A9 protein which having concentration of (2μM). After each addition of S10012, the sample was stirred for 2 min prior to scanning. Significant changes in emission spectrum were observed at 345 nm and the results were plotted as total concentration of S100A12 versus Relative intensity. The Eq 2 was utilized to fit a nonlinear curve in Graph-pad prism software and calculate the dissociation constant [38]:

$$\text{f}\;=\;[([{P]}_{T}+[{D]}_{T}+K_{d})-\sqrt{([{P]}_{T}+[{D]}_{T}+K_{d}{)}^{2}-4[{P]}_{T}[{D]}_{T}}\;]/2[{P]}_{T}$$ (2)

Where, f is the fractional change, *K*_*d*_ *is* the dissociation constant and [P] _T_ and [D] _T_ are the total concentration of S100A9 and S100A12 respectively.

## 3. Results and discussion

### 3.1 Binding interface of S100A9 and the S100A12

**Fig 1:**
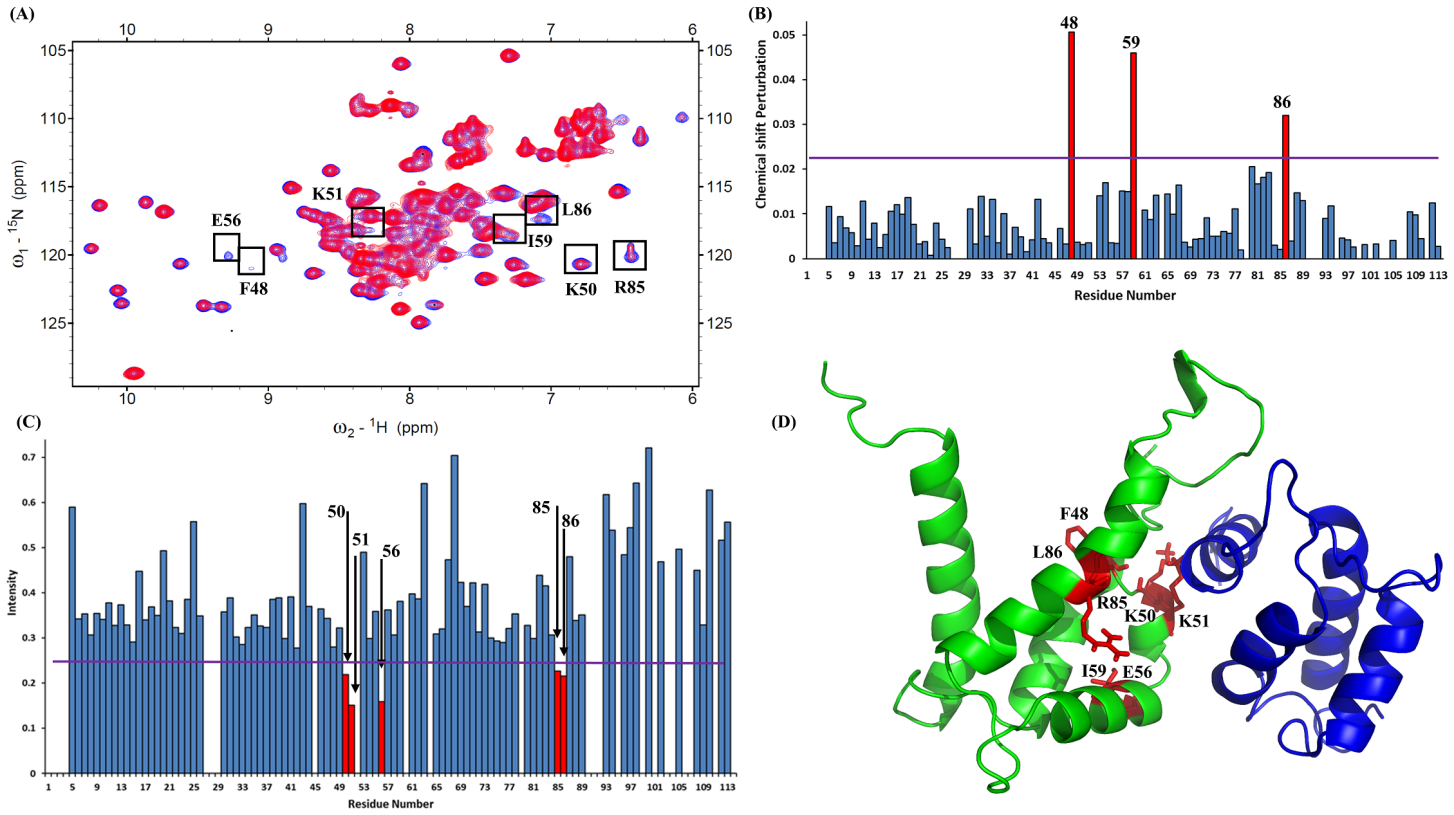
(A). Superimposed HSQC spectra of 1.01 mM S100A9 (^15^N-labeled) with those of S100A12 at molar ratios of 1:0 (blue) and 1:1 (red). The residues are shown in a black box were recognized using bar diagrams. (B). Bar diagram analysis of chemical shift (^1^H and ^15^N) perturbations of the amino acid residues in S100A12 upon complex formation with the S100A9. The threshold of selected residues exhibiting a significant change is represented by a violet line (>0.023). Following equation was used to calculated perturbation, combined shift difference = [(proton shifts)^2^ + (nitrogen shifts/6.51)^2^]^0.5^ (C). Bar diagram analysis of changes in cross-peak intensity ratio (I/I_0_). I represents the intensity of the S100A9–S100A12 complex, and I_0_ is the intensity of free S100A12. The violet line shows the threshold of selected residues that display a notably reduced intensity (<0.26). (D). Selected residues of S100A9 labeled in red on the three-dimensional structure of the heterodimer complex of S100A9 (green) and S100A12 (blue).

Two-dimensional (^1^H-^15^N HSQC) NMR spectroscopy is a convenient method for determining protein-ligand or protein-protein interactions [39]. This NMR technique gives substantial information for identifying the exchange regime of binding. The residues at the binding sites between S100A9 and S100A12 proteins were observed by superimposing the spectra of ^15^N-labeled S100A9 and that of ^15^N-labeled S100A9 complex with unlabeled S100A12 (Fig 1A). The interface region was identified based on changes in residue signals (either a decline in signal intensity or perturbation after complex formation originating from the residues at the interfaces of S100A9 and S100A12). The variation in the cross-peak chemical shift was used to correlate the spectra obtained with a protein—protein ratio of 1:0 and the complex spectra obtained with a ratio of 1:1. We used the following equation (Eq 3) to calculate the chemical shift variation [40]. Here ΔH, ΔN were showing proton shifts and nitrogen shifts respectively.

$$Chemical\;shift\;difference(\Delta \delta )\;=\;\sqrt{{\left(\Delta H\right)}^{2\;}+{\left(\frac{\Delta N}{6.51}\right)}^{2}}$$ (3)

On the other hand, I_0_ is the cross-peak intensity in the ^1^H-^15^N HSQC spectrum of ^15^N-labeled S100A9, while I_1_ is the cross-peak intensity of ^15^N-labeled S100A9 bound with the unlabeled S100A12 (intensity was monitored by Sparky). To discern the residues involvement in S100A9’s complex formation with S100A12, we created bar diagrams for convenient correlation of the HSQC titration results with observed the chemical shift perturbation (Fig 1B) and diminished intensity (Fig 1C). The bar diagram shows that most residues have decrease in cross-peak intensity. These seven residues are in the binding interface of the linker region (residue F48), helix-4 (residues K50 and K51), helix-5 (residues E56 and I59), and helix-6 (residues R85 and L86) (Fig 1D). And also these residues are shown as black boxes in the NMR spectrum (Fig 1A).

**Fig 2:**
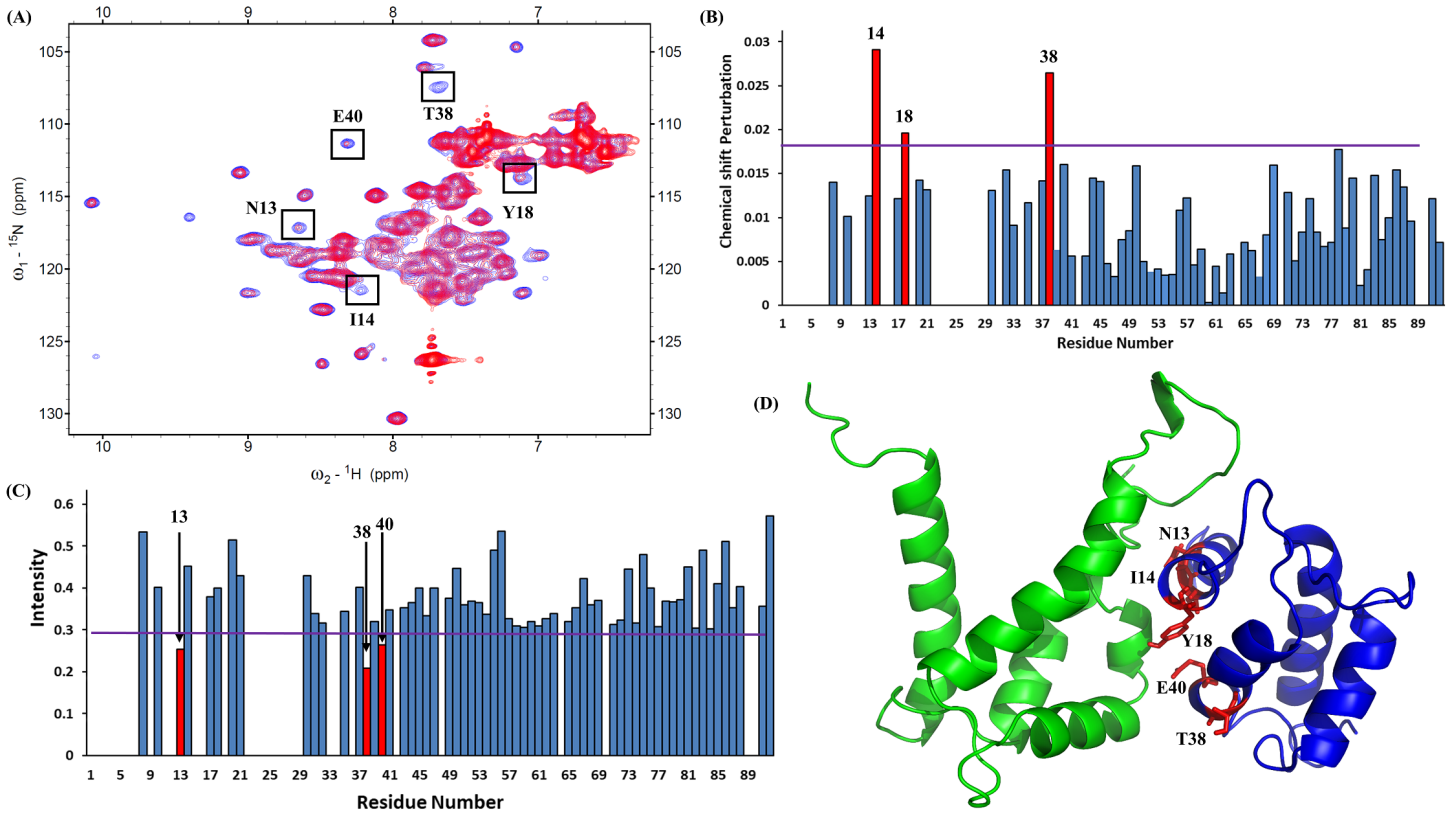
(A). Superimposed HSQC spectra of 0.98 mM S100A12 (^15^N-labeled) with those of S100A9 at molar ratios of 1:0 (blue) and 1:1 (red). These residues are shown in black box were recognized using bar diagrams. (B). Bar diagram analysis of chemical shift (^1^H and ^15^N) perturbations of the amino acid residues in S100A9 upon complex formation with the S100A12. The threshold of selected residues exhibiting significant changes was represented by a violet line (>0.017). Following equation was used to calculated perturbation, combined shift difference = [(proton shifts)^2^ + (nitrogen shifts/6.51)^2^]^0.5^ (C). Bar diagram analysis of changes in cross-peak intensity ratio (I/I_0_). I represent the intensity of the S100A12–S100A9 complex, and I_0_ is the intensity of free S100A12. The violet line shows the threshold of selected residues that display a notably reduced intensity (<0.29). (D). Selected residues of S100A12 labeled in red on the three-dimensional structure of the heterodimer complex of S100A12 (blue) and S100A9 (green).

Moreover, we have observed the superimposed spectra of the reverse HSQC titration. The experiment was done using the ^15^N-labeled S100A12 with unlabeled S100A9 at molar ratios of 1:0 and 1:1 (Fig 2A). We plotted a bar diagram to show the chemical shift perturbation (Fig 2B) and diminished intensity (Fig 2C). The diagram shows that some residues are perturbed and some are shifted. The five residues are on binding interfaces of helix-1 (residues N13 and I14), linker region (residue Y18), helix-2 (residues T38 and E40) (Fig 2D).

### 3.2 Docking studies

To investigate the protein-protein interactions, we used the HADDOCK platform to simulate a replica of the protein heterodimer complex. To accomplish this, the results of NMR HSQC titration were used to map the binding sites that appear in an area of S100A9 that forms a heterodimer structure with S100A12. The interaction restraints were obtained from the chemical shift perturbation or intensity change in the HSQC titration, which are the inputs for the HADDOCK computation. These restraints were mostly near each other, and the formation region of binding sites could be observed between S100A9 and S100A12.

Initially, we set up 2,000 complex structures that were generated by rigid-body minimization. Considering the total energy as the main criterion, the program refines the 200 best structures. Finally, the torsion angle and Cartesian dynamics were selected using a water model to compute various parameters of flexible structures and to find the best binding sites. Eventually HADDOCK divides these 200 structures into single clusters, from which we chose the best structures.

**Fig 3:**
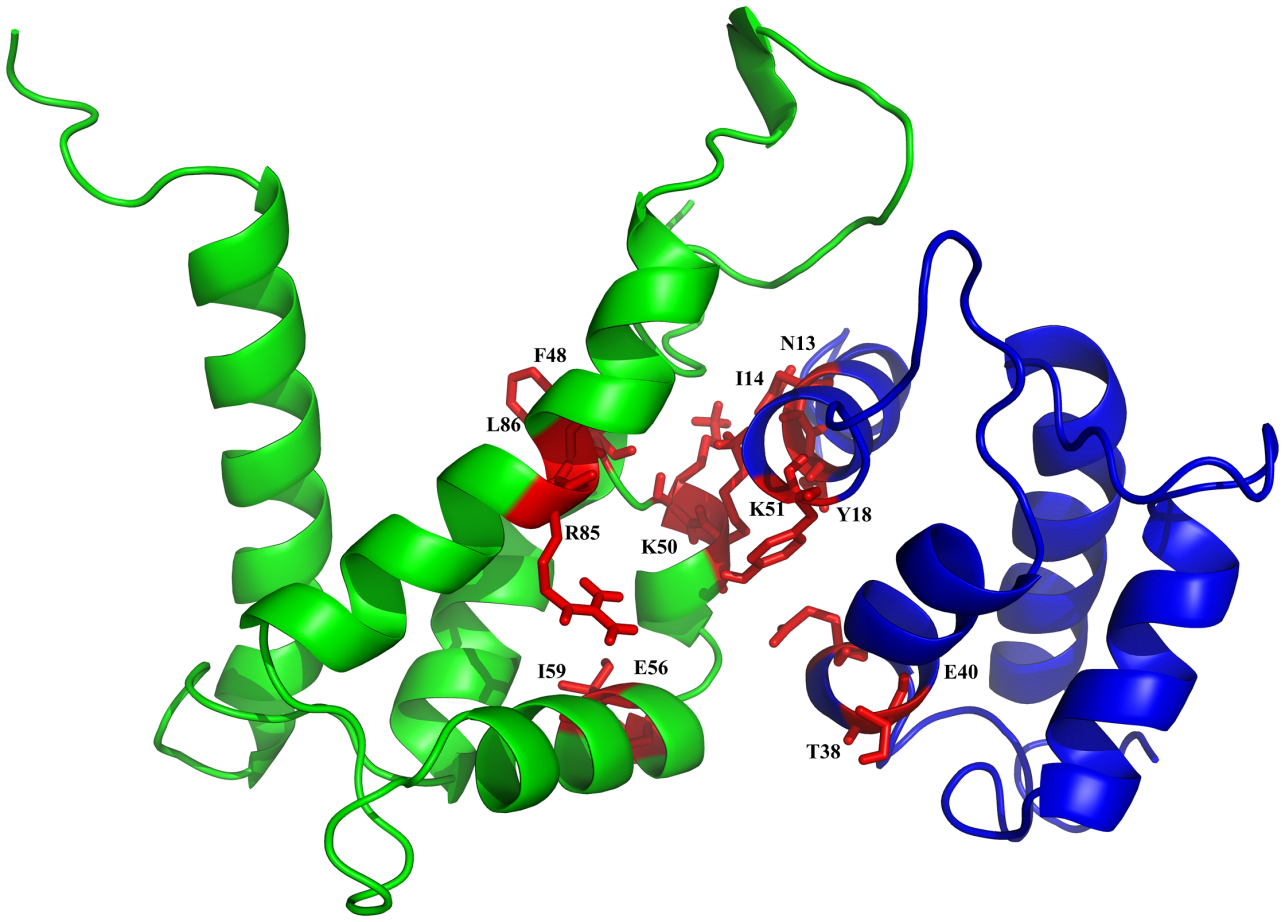
HADDOCK results shown as the binding site between S100A12 and S100A9 with side chains of selected residues. The stick structures of residues Asn-13, Ile-14, Tyr-18, Thr-38, and Glu-40 of S100A12 (blue) and of residues Phe-48, Lys-50, Lys-51, Glu-56, Ile-59, Agr-85, and Leu-86 of the S100A9 (green) are shown in red.

Fig 3 shows the mean structure of the heterodimeric S100A9-S100A12 complex with binding sites. The residues F48, K50, K51, E56, I59, R85, and L86 of S100A9 interacted with residues N13, I14, Y18, T38 and E40 of S100A12. PROCHECK analysis of the complex demonstrates reasonable stereochemistry for the bond angle and bond length [41]. A permissible percentage of 84% of the residues were allowed in the most favored region of the Ramachandran plot. There were only 1.0% of the residues near the disallowed region, and the corresponding average G-Factor score was 0.26. The results show that the usual region comprises an average structure (S5 Fig).

### 3.3 Binding constant measurements

The intrinsic fluorescence of tryptophan is sensitive to conformational changes of proteins and the polarity of the local environment where the protein associates with another protein. [42]. Tryptophan residues were exposed to emission maxima ranging from 308 to 350 nm with an excitation wavelength of 295 nm. However, the maximum emission of tryptophan residues is 308-330nm in hydrophobic environments [43].

**Fig 4:**
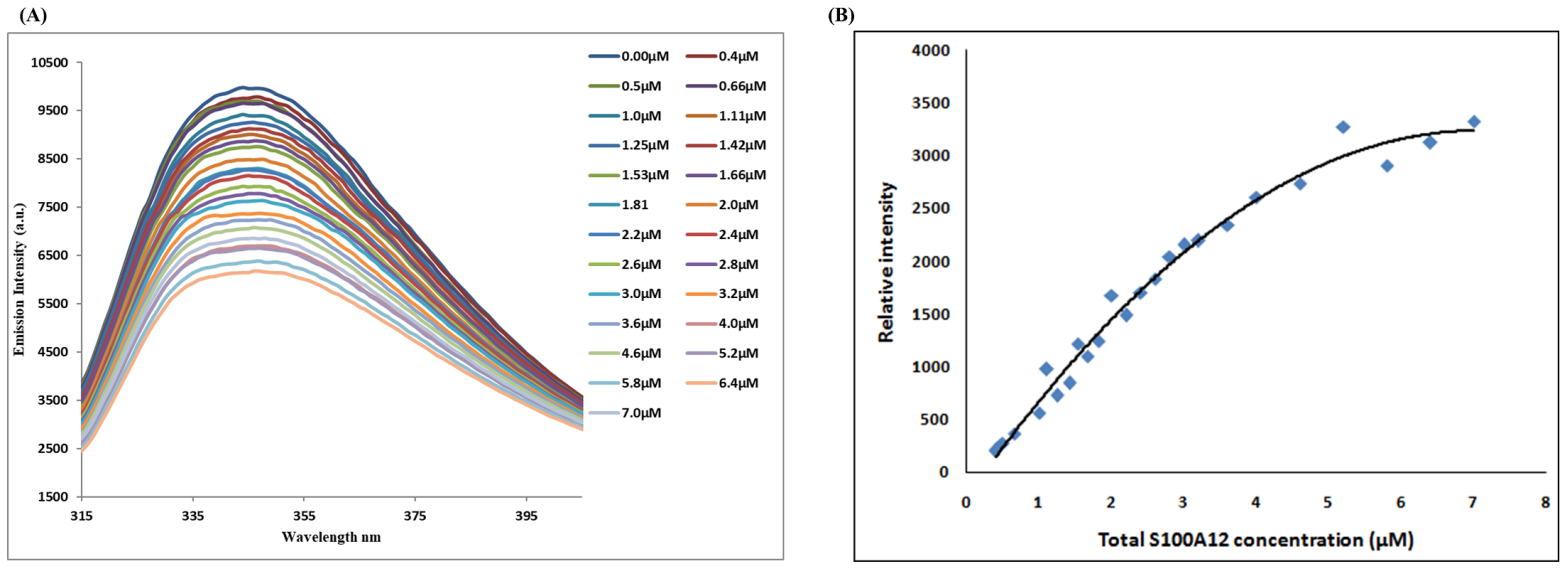
(A). Emission spectra of 2 μM S100A9 fluorescence titrations exhibiting decreased fluorescence intensities with increasing concentration of S100A12 at the micromolar level. (B). The nonlinear curve of fluorescence intensity changes versus S100A12 concentration at a wavelength of 345 nm. K_d_ was calculated as 1.3±0.36 μM using Eq 2.

There is one tryptophan residue in the S100A9 protein at Trp-88 position. The NACCESS result indicated that Trp-88 is the located on the interface of the S100A9. We observed the emission maxima of tryptophan residue at 345 nm upon excitation at 295 nm. The titration experiment shows decreasing fluorescence intensity of S100A9 protein with increasing S100A12 protein addition (Fig 4A). These results were curve fitted with the change in fluorescence intensity versus S100A12 concentrations in one binding site model (Fig 4B). We obtained a dissociation constant (K_d_) of 1.30±0.36. This was in the micromolar range, in the same way it’s indicate that formation of a stable S100A9-S100A12 protein complex under physiological conditions. A previous report showed that K_d_ for S100A9 binding with the RAGE V domain was 5–6 μM [28].

### 3.4 S100A12 inhibits the S100A9-RAGE V-domain complex

**Fig 5:**
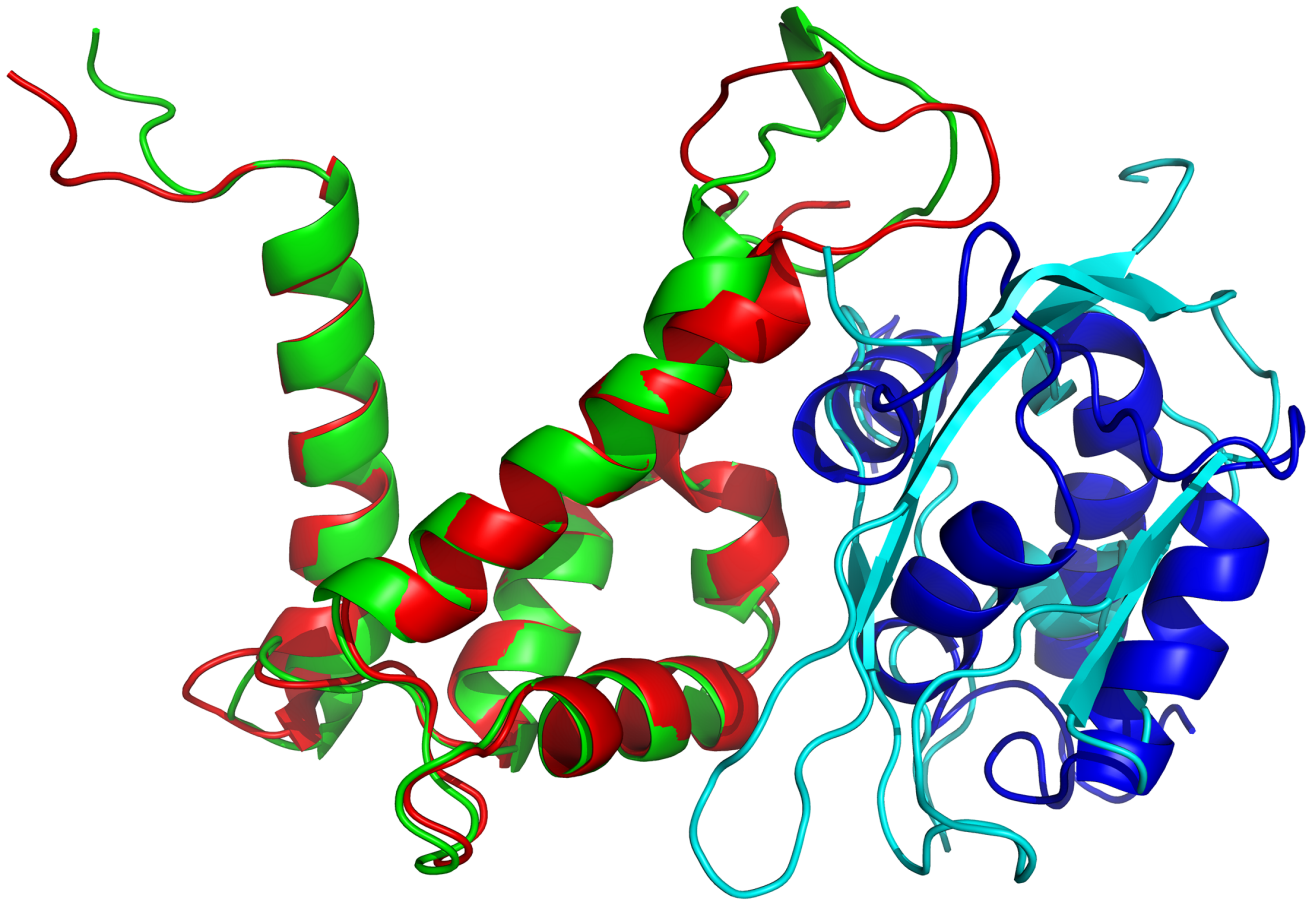
Superimposition of two complex structures (S100A9-RAGE and S100A9-S100A12). The S100A9-RAGE V-domain complex is shown in red and cyan, while the S100A9-S100A12 complex is shown in green and blue, respectively. The S100A12 protein clearly inhibits the binding between S100A9 and the RAGE V domain.

The interaction of S100A9 protein with the RAGE V domain leads to downstream signaling and triggers cell proliferation. Blocking the interactions between S100A9 and RAGE can prevent the cell proliferation activity [28]. After exam the S100A9 HSQC spectra with and without the unlabeled S100A12, the results show that residues of S100A9 protein were involved in the interaction with S100A12 protein based on perturbation and cross-peaks decreasing in the HSQC spectra of S100A9 (Fig 1A). The HADDOCK results suggest that binding region of S100A12 to S100A9 (Fig 5) significantly overlaps with the binding interface of the RAGE V-domain.

## 4. Discussion

RAGE is the most significant extracellular receptor and its interaction with S100 proteins can activate the transduction of cellular signals. This interaction is a noteworthy factor in the proliferation of cancer cells. A few studies have reported that the binding of the RAGE V domain and S100 proteins, such as S100P [44], S100A6 [45], and S100A9 [28] will induce cell proliferation. We now report the interactions between S100A12 and S100A9. With the help of an NMR HSQC titration and HADDOCK experiment, we demonstrated that binding occurs at the interface between the loop area of S100A9 and the hydrophobic area of S100A12. In Fig 5, the complex structure shows that S100A12 can blocks the binding interface of S100A9 and the V-domain of RAGE. A fluorescence titration experiment was used to measure the dissociation constants for the S100A9-S100A12 complex. The binding constant between S100A12 and S100A9 was in micromolar range. It means that S100A12 could be used as an antagonist of S100A9 against cell proliferation. Our analysis provides deep structural insight into the S100A9–S100A12 heterodimer complex structure and could favorable for the discovery of new drug development against cancer.

## Supporting information

S1 Fig Purified S100A12 protein shows a protein band corresponding to molecular weight of 10 kDa after SDS-PAGE. (TIF)

S2 Fig Purified S100A9 protein shows a protein band corresponding to molecular weight of 13 kDa after SDS-PAGE. (TIF)

S3 Fig ESI-MASS results confirm the molecular weight of purified S100A12. (TIF)

S4 Fig ESI-MASS results confirm the molecular weight of purified S100A9. (TIF)

S5 Fig Ramachandran plot statistics of the complex S100A9 with S100A12 using PROCHECK analysis. About 84% of the residues are in the favoured region and the disallow region is 0.5%. The overall average of G-factor is -0.2, which is in the usual region.
(TIF)
