# Supplementary figures and images for: Blocking the interaction between S100A9 protein and RAGE V domain using S100A12 protein
Source: PLoS One. 2018 Jun 14;13(6):e0198767. doi: 10.1371/journal.pone.0198767 (PMC6001950; doi:10.1371/journal.pone.0198767)

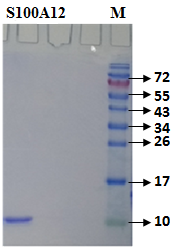

Supplement: S1 Fig — (TIF) [file pone.0198767.s001.tif]

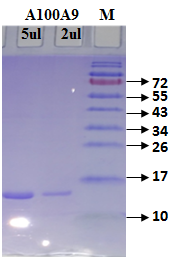

Supplement: S2 Fig — (TIF) [file pone.0198767.s002.tif]

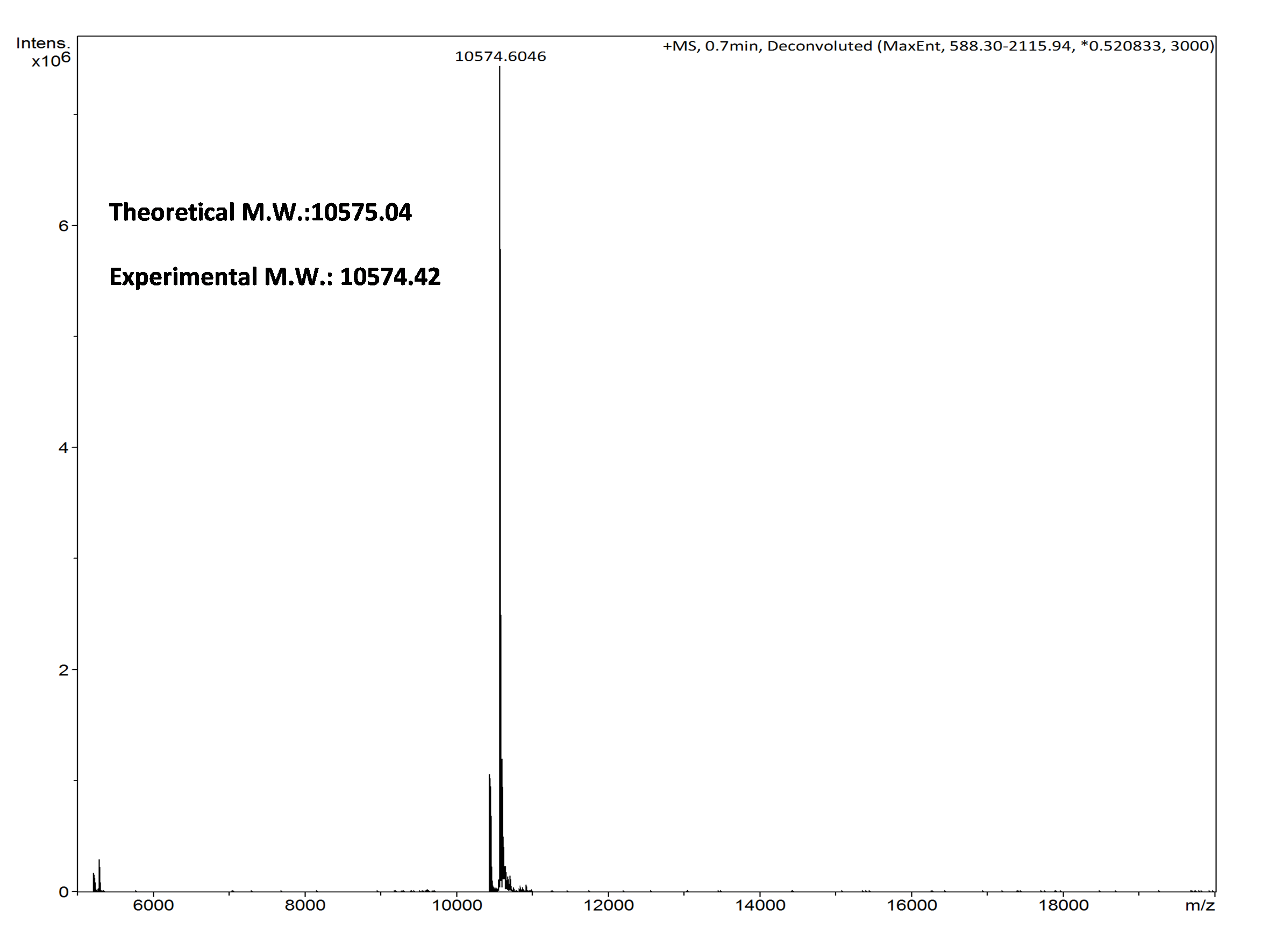

Supplement: S3 Fig — (TIF) [file pone.0198767.s003.tif]

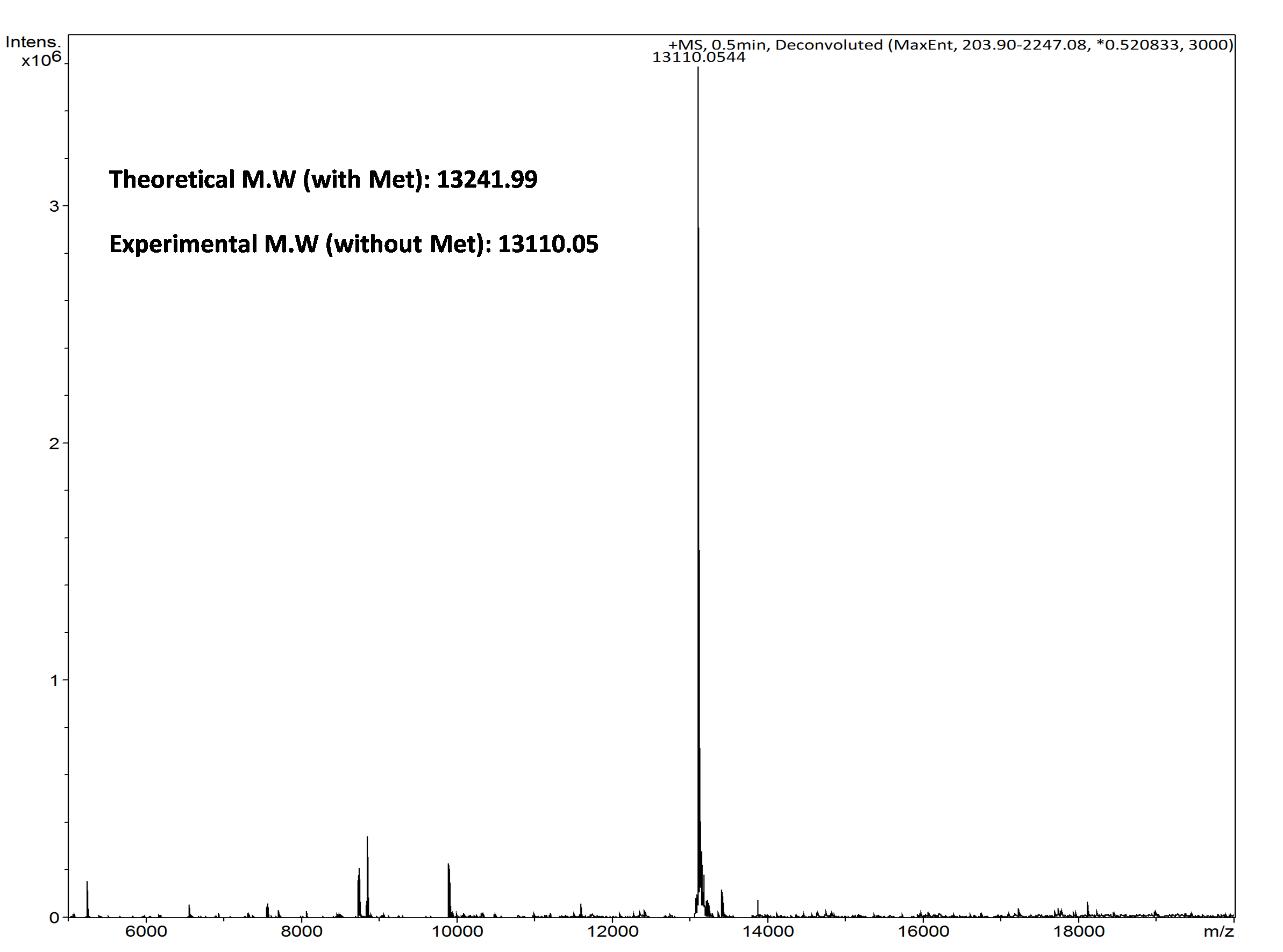

Supplement: S4 Fig — (TIF) [file pone.0198767.s004.tif]

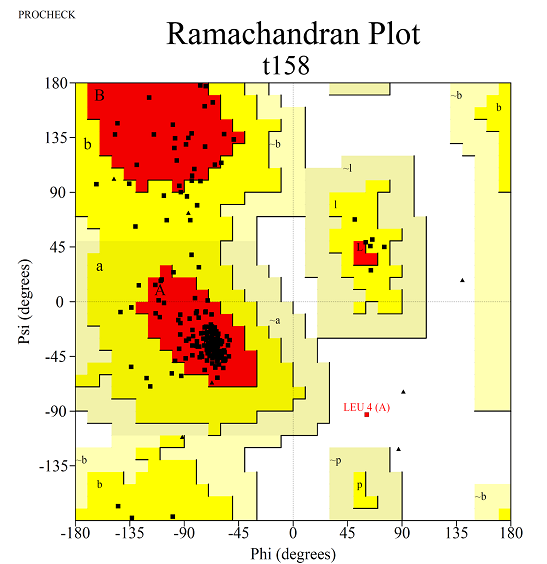

Supplement: S5 Fig — About 84% of the residues are in the favoured region and the disallow region is 0.5%. The overall average of G-factor is -0.2, which is in the usual region. (TIF) [file pone.0198767.s005.tif]
